# Braces versus Invisalign®: gingival parameters and patients’ satisfaction during treatment: a cross-sectional study

**DOI:** 10.1186/s12903-015-0060-4

**Published:** 2015-06-24

**Authors:** A. Azaripour, J. Weusmann, B. Mahmoodi, D. Peppas, A. Gerhold-Ay, C. J. F. Van Noorden, B. Willershausen

**Affiliations:** Department of Operative Dentistry, University Medical Center of the Johannes Gutenberg University Mainz, Augustusplatz 2, Mainz, 55131 Germany; Department of Cell Biology and Histology, Academic Medical Center, University of Amsterdam, Amsterdam, The Netherlands; Private practice, Mainz, Germany; Institute of Medical Biostatistics, Epidemiology and Informatics (IMBEI), Johannes Gutenberg University, Mainz, Germany

**Keywords:** Aligner, FOA, Braces, Dental hygiene, Periodontal health

## Abstract

**Background:**

Fixed orthodontic appliances (FOA) temporarily interfere with periodontal health of patients, as the appliance complicates oral hygiene. The use of aligners in orthodontic therapy increased strongly during the last decade. In the literature, the reports about effects of aligner treatment on oral hygiene and gingival conditions are scarce. This cross-sectional study evaluated oral hygiene and patient’s satisfaction during orthodontic treatment of patients with FOA or Invisalign®.

**Methods:**

100 patients (FOA = 50, Invisalign® = 50) were included who underwent orthodontic treatment for more than 6 months. Clinical examinations were performed to evaluate patients’ periodontal condition and were compared with clinical data at the beginning of the orthodontic treatment. Oral hygiene, patients’ satisfaction and dietary habits were documented by a detailed questionnaire. For statistical analysis, the Mann–Whitney U-Test and Fisher’s Exact Test were used; as multiple testing was applied, a Bonferroni correction was performed.

**Results:**

At the time of clinical examinations, patients with FOA were in orthodontic therapy for 12.9 ± 7.2 months, whereas patients with Invisalign® were in orthodontic therapy for 12.6 ± 7.4 months. Significantly better gingival health conditions were recorded in Invisalign® patients (GI: 0.54 ± 0.50 for FOA versus 0.35 ± 0.34 for Invisalign®; SBI: 15.2 ± 7.6 for FOA versus 7.6 ± 4.1 for Invisalign®), whereas the amount of dental plaque was also less but not significantly different (API: 37.7 % ± 21.9 for FOA versus 27.8 % ± 24.6 for Invisalign®). The evaluation of the questionnaire showed greater patients’ satisfaction in patients treated with Invisalign® than with FOA.

**Conclusion:**

Patients treated with Invisalign® have a better periodontal health and greater satisfaction during orthodontic treatment than patients treated with FOA.

**Electronic supplementary material:**

The online version of this article (doi:10.1186/s12903-015-0060-4) contains supplementary material, which is available to authorized users.

## Background

Fixed orthodontic appliances (FOA) promote the accumulation of bacterial plaque because FOA limit the ability of patients to perform good oral hygiene, which can lead to temporary destructive periodontal processes [1–4]. Deterioration of the periodontal status and dental decalcification during orthodontic treatment can be avoided only when the patient is incorporated in a stringent recall system [5, 6].

In the majority of patients, particularly during childhood and adolescence, FOA are the treatment of choice. Because of esthetics reasons, this treatment is not very popular for adult orthodontics. Therefore, other orthodontic techniques have been developed to increase esthetics and simplify oral hygiene procedures.

An alternative for FOA is Invisalign® which has been available since 1999 and offers not only the advantage of better esthetics but also the convenience of removal during food and beverage consumption, as well as oral care.

The Invisalign® system is a relatively novel treatment method and only a limited number of studies is available that compare effects of Invisalign® and FOA on oral hygiene. Miethke et al. [7, 8] showed that patients treated with Invisalign® do not have an increased periodontal risk, although both teeth and gingiva were covered nearly the entire day with aligners. It indicates that patients using the Invisalign® system have a better periodontal health as compared to FOA-treated patients.

The aim of the present study was to compare oral health status, oral hygiene and patients satisfaction during orthodontic treatment of patients with FOA and Invisalign®.

The primary hypothesis of this study was that Invisalign® patients have better oral hygiene and gingival inflammation parameters than FOA patients. Second hypothesis was that Invisalign® is associated with a better quality of life during orthodontic treatment than FOA.

## Methods

### Inclusion of patients

A total of 139 consecutive orthodontic patients were screened for this cross-sectional study.

The following patient inclusion criteria were applied:

- FOA or Invisalign® for at least six months;
- Modified sulcus bleeding index (SBI) ≤20 % [9] prior to orthodontic treatment;
- Approximal plaque index (API) ≤25 % [9] prior to orthodontic treatment;
- Declaration of consent.

Exclusion criteria were:

- History of periodontitis;
- Diseases that affect periodontal health;
- Smoking;
- Pregnancy;
- Withdrawal of consent;
- Participation in another clinical trial.

All patients received the same oral hygiene instructions before and during orthodontic treatment. This included the proper use of toothbrush, dental floss and interdental brushes. Patients were recommended to use all three measures of oral care three times daily. Furthermore, after periodontal examinations, patients received professional hygiene prophylaxis treatment prior to orthodontic treatment, and were also enrolled in a recall system including professional cleaning every six months.

### Screening visit

Participants and, in case that they were minors, their parents or guardians were informed both verbally and in writing about the possibility of their data being used anonymously for study purposes and the data privacy regulations. The subjects signed the informed consent form when they accepted to participate voluntarily in the clinical trial. Furthermore, retrospective data from voluntary participants were collected from the baseline visit (prior to the orthodontic treatment).

### Approval

The study was approved by the medical ethics committee of the Johannes Gutenberg University Medical Center (837.304.13 (8989)).

### Clinical examination before and during orthodontic treatment

One calibrated examiner performed all oral examinations. Since it was easy to distinguish FOA from Invisalign® by oral inspection, no blinding was possible during treatment.

In all patients, the gingival condition was evaluated using the gingiva index (GI) of Silness and Löe [10], and SBI according to Lange et al. [9].

For the Invisalign® patients, the API according to Lange et al. [9] was assessed. For FOA patients, the amount of plaque was recorded using the modified plaque index (MPI) according to Attin et al. [11] and adjusted to API. Plaque disclosing tablets (Produits Dentaires S.A., Vevey, Switzerland) were used for 30 seconds to assess API and MPI.

### Quality-of-life questionnaires

All patients participating in the clinical examination were questioned about their overall well-being, whether they would be willing to undergo the same treatment again, oral hygiene habits, food choices and the frequency and method of toothbrushing using a specially designed quality-of-life questionnaire (Additional file 1).

### Statistical analysis

The statistical analysis was carried out using SPSS 22.0 (SPSS Inc., Chicago, IL, USA). Descriptive statistics were calculated for all used variables. For the categorical data absolute and relative frequencies are presented, for the continuous data mean and standard deviation are shown.

The major aims of this study are the investigation of differences in periodontal parameters API, SBI and GI between patients with FOA and Invisalign®. To address these aims, we applied a linear mixed model including age as covariate for API, SBI and GI, respectively. Bonferroni correction with a local significance level of α = 0.016 was used to account for multiple testing.

All other analyses were performed exploratively. Mann–Whitney U-Test was used for continuous data and Fisher’s Exact Test was used for categorical variables. All p-values were given only for description and should be interpreted with caution.

## Results

### Patient characteristics

A total of 100 orthodontic patients met the inclusion criteria and were enrolled. The age of the patients varied from 11 to 62 years. Fifty patients received treatment with Invisalign® (11 male and 39 female) and 50 with FOA (16 male and 34 female). The patients in the FOA group (n = 50) were on average 16.3 ± 6.9 years old with an age range of 11–61 years. The Invisalign® patients (n = 50) were on average 31.9 ± 13.6 years of age with a range of 12–61 years. The majority of the participants was female (FOA: 68 %, Invisalign®: 78 %). Orthodontic treatment was performed for 12.9 ± 7.2 months in FOA patients and 12.6 ± 7.4 months in Invisalign® patients at the time of the screening visit (Table 1).

**Table 1:** Demographic data of patients with FOA or Invisalign® included in the study

Variable	FOA	Invisalign®	p-value
Age (years)	16.3 ± 6.9	31.9 ± 13.6	≤0.001
Gender (male/female)	16 / 34	11 / 39	0.260
Duration of orthodontic treatment (months)	12.9 ± 7.2	12.6 ± 7.4	0.820

Values represent descriptive mean ± standard deviation

### Clinical outcomes

#### Prior to orthodontic treatment (baseline visit)

The analysis of the data showed no differences in periodontal conditions prior to the orthodontic treatment. Both groups had implemented good oral hygiene as measured by API: 19.6 % ± 7.0 (FOA patients) and 16.3 % ± 9.6 (Invisalign® patients) and had good periodontal health (Table 2).

**Table 2:** Clinical parameters of patients with FOA or Invisalign® before and during orthodontic treatment

Clinical parameter	FOA	Invisalign®	p-value
API (%) - before treatment	19.6 ± 7.0	16.3 ± 9.6	0.068
API (%) - during treatment	37.7 ± 21.9	27.8 ± 24.6	0.108
Relative difference	18.1 ± 17.2	11.5 ± 17.2	0.170*
SBI (%) - before treatment	7.2 ± 4.4	6.6 ± 3.2	0.503
SBI (%) - during treatment	15.2 ± 7.6	7.6 ± 4.1	≤0.001
Relative difference	8.0 ± 6.6	1.0 ± 1.0	≤0.001*
GI - before treatment	0.29 ± 0.24	0.27 ± 0.25	0.910
GI - during treatment	0.54 ± 0.50	0.35 ± 0.34	0.072
Relative difference	0.25 ± 0.30	0.07 ± 0.12	0.001*

Values represent descriptive mean ± standard deviation

*Adjusted for age

#### Screening visit during orthodontic treatment

There were notable changes in periodontal conditions in both groups during orthodontic treatment. Dental plaque measured by API or MPI had increased in both groups but was higher in the FOA patients (37.7 % ± 21.9) as compared to the Invisalign® patients (27.8 % ± 24.6). These differences were not significant. Invisalign® patients showed significantly better gingival conditions than FOA patients. GI and SBI values were hardly increased in Invisalign® patients during orthodontic treatment whereas GI and SBI values increased 2-fold in FOA patients during orthodontic treatment (Table 2).

### Subjective data obtained with quality-of-life questionnaire

The quality-of-life questionnaire revealed that Invisalign® patients reported less impairment on general well being as compared to FOA patients (6 % versus 36 %, p = 0.001). More FOA patients than Invisalign® patients reported to suffer from laughing inhibition because of esthetics (26 % versus 6 %, p = 0.012), whereas 98 % of the Invisalign® patients would be willing to undergo the same treatment again, and 78 % of the FOA patients (p = 0.004). Furthermore, 70 % of FOA patients compared to only 50 % of Invisalign® patients reported that their eating habits had changed during orthodontic treatment (p = 0.066). FOA-treated patients reported more frequently to have to brush their teeth more often than before the start of the orthodontic treatment (84 % FOA patients versus 52 % Invisalign® patients, p = 0.001), whereas approx. 50 % of the patients in both groups used an electric toothbrush (Table 3). The FOA patients reported more gingiva irritation in comparison to the Invisalign® patients (FOA: 56 %; Invisalign®: 14 %; p = 0.001).

**Table 3:** Subjective data of patients with FOA or Invisalign® during orthodontic treatment

Variable	FOA	Invisalign®	p-value
Impairment of general well being	36	6	0.001
Suffer under laugh inhibition	26	6	0.012
Would decide again to undergo the same treatment	78	98	0.004
Change of eating habits	70	50	0.066
Increased frequency of tooth brushing	84	52	0.001
Electric toothbrush	50	46	0.841
Subjective gingiva irritation (% yes)	56	14	0.001
Brushing time (min)	3.7 ± 1.7	2.2 ± 1.2	0.001
Change of toothbrush (in months)	2.2 ± 1.2	2.7 ± 1.2	0.020

Values represent relative frequency of individuals’ positive responses (%) and descriptive mean ± standard deviation (last two questions)

Table 3 shows that FOA patients spent 3.7 ± 1.7 min on average as tooth-brushing time with a minimum of one min and a maximum of 15 min, whereas Invisalign® patients reported to brush their teeth on average during 2.2 ± 1.2 min with a minimum of 1.5 min and a maximum of up to 8 min. FOA patients did not change their toothbrush as frequently as Invisalign® patients.

## Discussion

We found that orthodontic treatment has less negative impact on Invisalign® patients than on FOA patients with respect to both gingival condition and patient well being. We did not find any significant plaque accumulation in both patient groups.

It has been shown that FOA can lead to increased plaque accumulation and reduced oral hygiene during orthodontic treatment [12, 13]. Various studies compared different orthodontic approaches and it was shown that removable appliances caused less plaque accumulation and better oral health [7, 8, 14].

Increased plaque accumulation [3, 4, 15] can lead to gingival inflammation, increased susceptibility to caries, decalcifications or white spot lesions [15–17]. Miethke et al. showed that the plaque index was significantly lower in patients treated with Invisalign® than in FOA patients, but that other periodontal conditions in both groups were similar [7]. Due the introductory training provided for oral hygiene and instructions for optimal tooth brushing, all patients in our study were very cooperative. The majority of patients used the regular recall appointments and put great emphasis on dental esthetics. This may be a reason why we did not find a significant difference in plaque accumulation between both patient groups.

In contrast, gingival inflammation was significantly lower in the Invisalign® patients whereas Miethke et al. reported significant differences only inside the groups. In a later study, Miethke et al. compared the gingival status in orthodontic patients treated with Invisalign® or lingual brackets and found that periodontal parameters were worse in patients with lingual brackets [8].

Although the majority of study participants had a satisfactory oral hygiene, the difference in time needed for brushing teeth was clearly shorter in Invisalign® patients than in FOA patients. This difference is certainly due to removal of Invisalign® aligners which facilitates an easier and faster tooth cleaning.

Borutta et al. found that in patients with FOA, electric toothbrushes gave better oral hygiene results than manual brushing. A significantly better plaque removal and reduction of gingival inflammation was observed when tooth brushing was done manually [16]. In contrast, Hickman et al. [15] and Deery et al. [17] observed no significant differences between the two tooth brushing systems. In our study, the patients’ toothbrush preferences were similar in both treatment groups.

Sergl et al. [18] described the impairment of everyday life as a result of orthodontic treatment. Bernabé et al. showed that there was significantly greater impact on the daily life of patients with FOA as compared to patients with removable appliances [19]. In their study, impairments in speech and eating habits were especially noted in patients who were in the 15–16 years age category.

It must be mentioned that in our study the average age of the FOA and Invisalign® patients was notably different; the FOA group mainly consisted of teenagers and young adults whereas the Invisalign® group consisted primarily of adults. Therefore, we used linear regression models for our confirmatory questions and adjust for age to exclude the effect of age on the outcome of our study.

## Conclusions

Our primary hypothesis has been largely confirmed. Invisalign® patients have significantly better gingival health, whereas oral hygiene is not different between FOA patients and Invisalign® patients. Our second hypothesis that Invisalign® is superior for quality of life of the patients has also been confirmed.

Finally, Invisalign® is more gentle for gingival tissue than FOA due to more simple oral hygiene.

## Additional file

Additional file 1:
**Patient questionnaire.**

## Abbreviations

API: Approximal plaque index
FOA: Fixed orthodontic appliances
GI: Gingiva index
MPI: Modified plaque index
SBI: Sulcus bleeding index
