# Supplementary material for: Braces versus Invisalign®: gingival parameters and patients’ satisfaction during treatment: a cross-sectional study
Source: BMC Oral Health. 2015 Jun 24;15:69. doi: 10.1186/s12903-015-0060-4 (PMC4478712; doi:10.1186/s12903-015-0060-4)
Supplement: Additional file 1: — Patient questionnaire. [file 12903_2015_60_MOESM1_ESM.doc]

Appendix 1

**Patient questionnaire**

1. How often do you brush your teeth per day? ☐ more than 2x per day

☐ 2x per day

☐1x per day

☐ never

2. How do you brush your teeth? ☐ Hand brush

☐ Electric brush

3. Do you use a mouth rinse? ☐ more than 2x

☐ 2x per day

☐ 1x per day

☐ never

4. Do you use interdental brushes? ☐ more than 2x

☐ 2x per day

☐ 1x per day

☐ never

5. Do you use a dental floss? ☐ more than 2x

☐ 2x

☐ 1x

☐ never

6. How many minutes do you brush your teeth each time? ....... minutes

7. Do you always brush your teeth after meals ☐ Yes

☐ No

8. How often do you change your toothbrush (in months)? ..…

9. It is difficult to brush my teeth appropriately ☐Yes

☐ No

If yes, why? ☐ Crowding

☐ Hand movement

☐ Mouth opening

10. I have to brush more often than before treatment ☐ Yes

☐ No

11. I need additional aids to brush appropriately ☐ Yes

☐ No

12. I got gingival problems like swelling, redness or bleeding due to treatment ☐ Yes

☐ No

13. Does the appliance have an impairment on your general well-being? ☐Yes

☐ No

14. Does the appliance influence your everyday life? ☐ Yes

☐ No

If yes, when? ☐ Meals

☐ Speaking

☐ Sports

☐ Sleep

☐ At work

15. How do you estimate your own oral hygiene since you got the appliance? ☐ very good

☐ good

☐ average

☐ bad

16. How can you handle the appliance? ☐ very good

☐ good

☐ average

☐ bad

17. Did you suffer from headache prior to therapy? ☐ Yes

☐ No

18. Did this improve under therapy? ☐ Yes

☐ No

If yes, since how many months? .......

19. Did you suffer from neck pain prior to therapy? ☐ Yes

☐ No

20. Did this improve under therapy? ☐ Yes

☐ No

If yes, since how many months? ..................................

21. Did you suffer from back pain prior to therapy? ☐ Yes

☐ No

22. Did this improve under therapy? ☐ Yes

☐ No

If yes, since how many months? ...........

23. Did you suffer from cranio-mandibular problems prior to therapy? ☐ Yes

☐ No

24. Did this improve under therapy? ☐ Yes

☐ No

If yes, since how many months? ..........

25. Did you suffer from tinnitus prior to therapy? ☐ Yes

☐ No

26. Did this improve under therapy? ☐ Yes

☐ No

If yes, since how many months? ..................................

27. Did your eating habits change under therapy ☐ Yes

☐ No

28. Do you feel an impact towards your outer appearance by the device? ☐ Yes

☐ No

29. Do you feel an inhibition to laugh heartily? ☐ Yes

☐ No

30. Would you choose the same therapy again? ☐ Yes

☐ No
